# Fatty acids and inflammatory stimuli induce expression of long-chain acyl-CoA synthetase 1 to promote lipid remodeling in diabetic kidney disease

**DOI:** 10.1016/j.jbc.2023.105502

**Published:** 2023-11-26

**Authors:** Chih-Hong Wang, Surbhi, Sayhaan Goraya, Jaeman Byun, Subramaniam Pennathur

**Affiliations:** 1 Department of Physiology, Tulane University of School Medicine, New Orleans, Louisiana, USA; 2 Tulane Hypertension & Renal Center of Excellence, Tulane University, New Orleans, Louisiana, USA; 3 Division of Nephrology, Department of Internal Medicine, University of Michigan, Ann Arbor, Michigan, USA; 4 Department of Molecular and Integrative Physiology, University of Michigan, Ann Arbor, Michigan, USA

**Keywords:** ACSL1, diabetic kidney disease, inflammation, lipid, metabolomics

## Abstract

Fatty acid handling and complex lipid synthesis are altered in the kidney cortex of diabetic patients. We recently showed that inhibition of the renin-angiotensin system without changes in glycemia can reverse diabetic kidney disease (DKD) and restore the lipid metabolic network in the kidney cortex of diabetic (*db/db)* mice, raising the possibility that lipid remodeling may play a central role in DKD. However, the roles of specific enzymes involved in lipid remodeling in DKD have not been elucidated. In the present study, we used this diabetic mouse model and a proximal tubule epithelial cell line (HK2) to investigate the potential relationship between long-chain acyl-CoA synthetase 1 (ACSL1) and lipid metabolism in response to fatty acid exposure and inflammatory signals. We found ACSL1 expression was significantly increased in the kidney cortex of *db/db* mice, and exposure to palmitate or tumor necrosis factor–α significantly increased *Acsl1* mRNA expression in HK-2 cells. In addition, palmitate treatment significantly increased the levels of long-chain acylcarnitines and fatty acyl CoAs in HK2 cells, and these increases were abolished in HK2 cell lines with specific deletion of *Acsl1(Acsl1*KO cells), suggesting a key role for ACSL1 in fatty acid β-oxidation. In contrast, tumor necrosis factor–α treatment significantly increased the levels of short-chain acylcarnitines and long-chain fatty acyl CoAs in HK2 cells but not in *Acsl1*KO cells, consistent with fatty acid channeling to complex lipids. Taken together, our data demonstrate a key role for ACSL1 in regulating lipid metabolism, fatty acid partitioning, and inflammation.

Diabetic kidney disease (DKD) is a progressive, microvascular complication of diabetes mellitus that results in impaired renal function and is the leading cause of end-stage renal disease worldwide (1). While previous research on DKD has focused on glomerular pathology, such as podocyte loss and glomerulosclerosis, growing evidence indicates that metabolic dysfunction in the renal tubules plays an important role in the progression of DKD (2). Lipid accumulation in the renal tubules, an indicator of metabolic syndrome, leads to defective fatty acid oxidation in renal tubular epithelial cells and tubulo-interstitial fibrosis (2, 3). Furthermore, the lipotoxicity induced by excessive lipid accumulation promotes impaired renal function by inducing hypoxia, mitochondrial dysfunction, chronic inflammation, and renal fibrosis (4, 5, 6).

Dyslipidemia is a major risk factor for the development and progression of DKD (7, 8). Our previous studies demonstrated that fatty acid handling and complex lipid synthesis are significantly altered in the diabetic kidney and are distinct from that observed in other complication-prone tissues, such as the nerves and retina, consistent with tissue-specific remodeling (9). Importantly, inhibition of the renin-angiotensin system, an intervention that reverses DKD without altering glycemic control, restored the lipid metabolic network in the proximal tubules of diabetic *db/db* mice, raising the possibility of a direct role of renal lipid metabolism in DKD progression, independent of glycemia (9). Additionally, the administration of lipid-lowering agents improved kidney function in animal models of DKD as evidenced by reduced proteinuria and renal fibrosis (10, 11, 12). Indeed, clinical studies have also shown protective effects of lipid-lowering drugs in patients with DKD. For example, statins, which are 3-hydroxy-3-methyl-glutaryl-coenzyme A reductase inhibitors, attenuated the development of DKD in patients with type 2 diabetes (13, 14). Further, proprotein convertase subtilisin/kexin type 9 inhibitors that facilitate low-density lipoprotein uptake and clearance by hepatocytes ameliorated the hyperlipidemia and renal dysfunction caused by nephrotic syndrome (15). Together, these findings suggest that further understanding of the mechanisms underlying lipid metabolism in DKD may lead to novel therapeutics approaches.

Long-chain acyl-CoA synthetase 1 (ACSL1) plays an important role in lipid metabolism by promoting fatty acid oxidation and is highly expressed in most energy-demanding cells in the body, including renal proximal tubule cells (16). ACSL1 catalyzes the conversion of free long-chain fatty acids to fatty acyl-CoAs prior to β-oxidation, and thereby reduces glucose utilization. ACSL1 deficiency impairs fatty acid oxidation and results in increased glucose metabolism and lipid accumulation (17). Moreover, ACSL1 is a direct target gene of peroxisome proliferator-activated receptor (PPAR)α and PPARγ in liver and adipose tissue, respectively (18, 19, 20). These findings indicate an important link between PPARα and PPARγ signaling pathways and the divergent functional roles of ACSL1 in different tissues, including renal lipid metabolism. Although ACSL1 clearly plays an important role in lipid metabolism, insulin resistance, and obesity, no studies so far have investigated the association between ACSL1 and DKD. Therefore, we explored the potential relationship between ACSL1 and DKD.

In the present study, we investigated the potential link between ACSL1 and DKD in diabetic (*db/db*) mice and in a proximal tubule epithelial cell line (HK2 cells). We found that ACSL1 was upregulated in the kidney cortex of *db/db* mice, concomitant with the upregulation of well-known inflammatory mediators. Furthermore, targeted deletion of *Acsl1* in HK2 cells inhibited the upregulation of proinflammatory genes and long-chain fatty acids observed in HK2 cells following palmitate (PA) or tumor necrosis factor (TNF)-α treatment. These results revealed an important role of ACSL1 in regulating the lipid metabolism and inflammation in proximal tubule cells in DKD and suggest ACSL1 as a potential therapeutic target for the treatment of DKD.

## Results

### ACSL1 expression is upregulated in diabetic *db/db* mice

**Figure 1:**
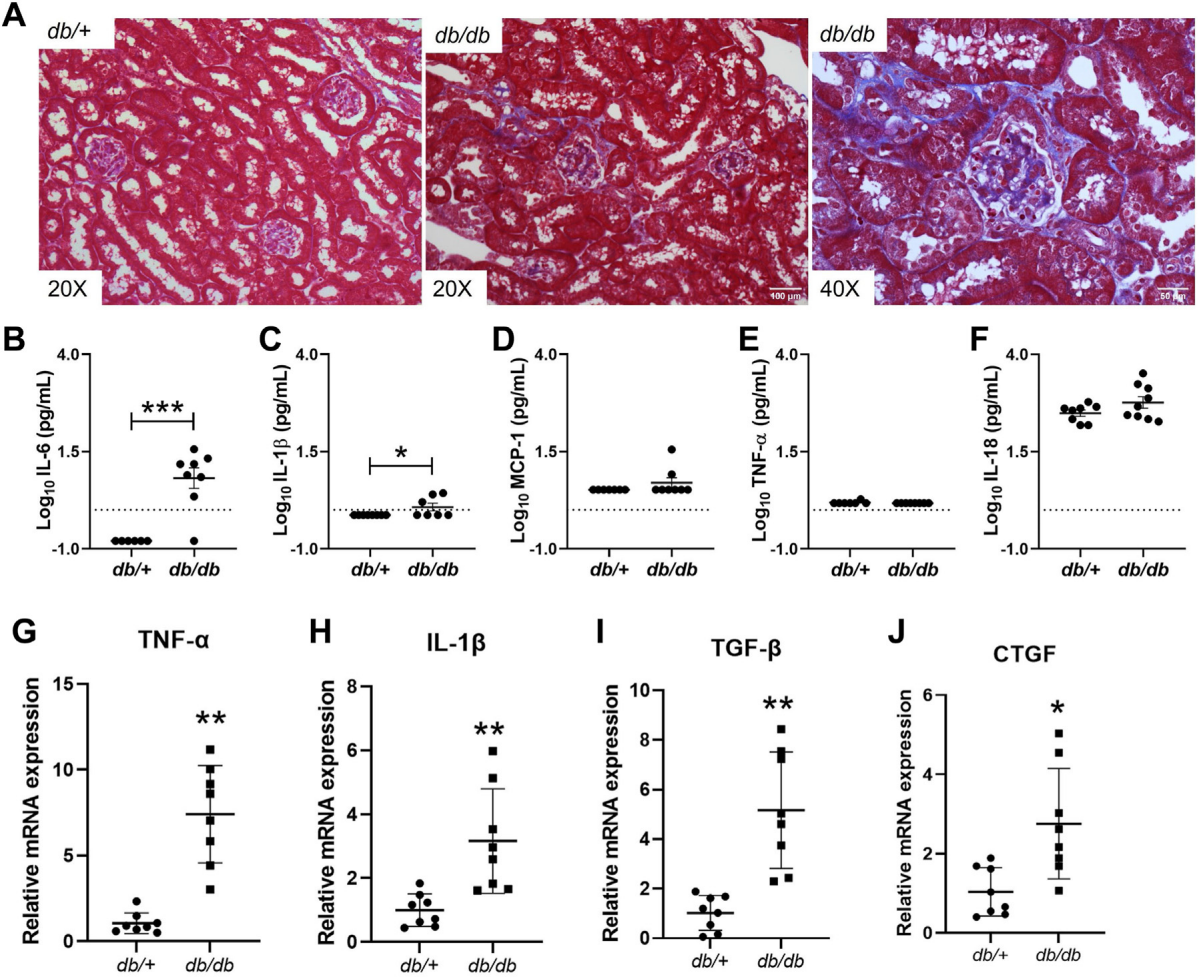
**Diabetic *db/db* mice exhibited increased renal fibrosis and inflammation as compared with *db/+* mice.** To confirm the DKD development, we examined the degree of fibrosis, plasma proinflammatory cytokine levels, and mRNA levels of inflammatory markers in the renal cortex of 24-week-old *db/db* mice. *A,* Masson's trichrome staining in renal cortex sections from *db/+* and *db/db* mice (n = 3/group). *B–F,* plasma proinflammatory cytokine levels in *db/+* and *db/db* mice (n = 8/group): *B,* IL-6; *C,* IL-1β; *D,* MCP-1; *E,* TNF-α; and *F,* IL-18. Statistical differences in cytokines expression were assessed using a two-tailed unpaired student’s *t* test. *G–J,* relative mRNA levels of inflammatory markers in *db/+* and *db/db* mice (n = 8/group), *G*, TNF-α and *H*, IL-1β, and fibrosis-related genes. *I*, TGF-β and *J*, CTGF. Statistical differences in expression levels of inflammatory and fibrosis markers were assessed using a two-tailed unpaired student’s *t* test. Data are shown as mean ± SD. ∗*p* < 0.05 and ∗∗*p* < 0.01. CTGF, connective tissue growth factor; DKD, diabetic kidney disease; IL, interleukin; MCP-1, monocyte chemoattractant protein-1; TGF, tumor growth factor; TNF, tumor necrosis factor.

The *db/db* mouse model is a well-established model of type 2 diabetes mellitus. These mice develop DKD, accompanied by hyperlipidemia and inflammation, by 24 weeks of age (18). First, we confirmed that DKD developed in these mice by examining the degree of fibrosis in the renal cortex of *db/db* mice and plasma proinflammatory cytokine levels. By 24 weeks of age, *db/db* mice developed tubulointerstitial fibrosis and showed increased plasma levels of proinflammatory cytokines, including interleukin IL-Iβ and IL-6, but no differences in monocyte chemoattractant protein-1, TNF-α, and IL-18 (Fig. 1, *A*–*F*). The mRNA expression of proinflammatory cytokines (TNF-α and IL-1β) and renal fibrosis markers (tumor growth factor [TGF]-β1 and connective tissue growth factor) were also consistently upregulated in the renal cortex of 24-week-old *db/db* mice (Fig. 1, *G*–*J*).

**Figure 2:**
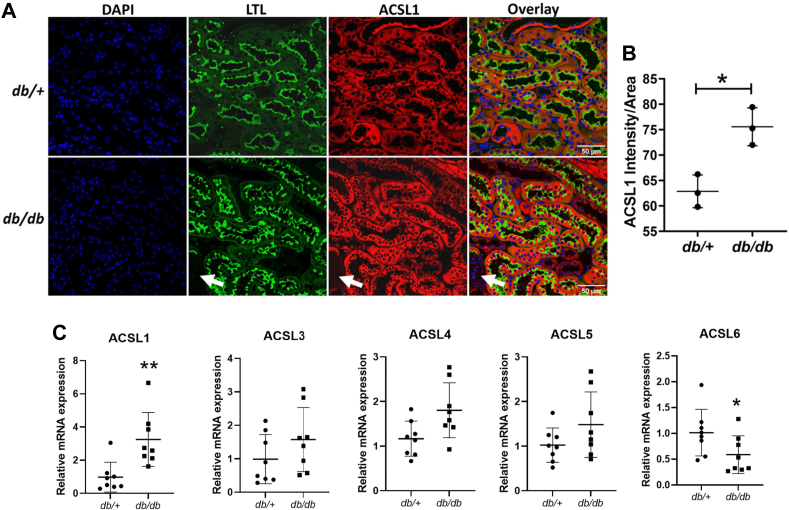
**Upregulated ACSL1 expression in the proximal tubules in *db/db* mice.** To examine whether ACSL expression was altered in the renal cortex of *db/db* mice, we quantified the ACSL1 protein and mRNA expression in the renal proximal tubules in *db/db* mice. *A,* confocal images showing ACSL1 protein expression in the renal proximal tubules in *db/+* and *db/db* mice (n = 3/group). *Arrow* indicates the absence of ACSL1 expression in glomeruli. *B,* quantification of ACSL1 protein expression (intensity/area) in microscopic images from *db/+* and *db/db* mice (two images were captured from renal cortex and ten proximal tubules were quantified for each mouse). *C,* relative mRNA expression of ACSL isoforms: *Acsl1*, *Acsl3*, *Acsl4*, *Acsl5*, *Acsl6*, in the renal cortex of *db/+* and *db/db* mice (n = 8/group). Statistical differences in ACSL1 protein and mRNA expression levels were assessed using a two-tailed unpaired student’s *t* test. Data are shown as mean ± SD. ∗*p* < 0.05 and ∗∗*p* < 0.01. ACSL, acyl-CoA synthetase.

Further, to examine whether ACSL expression was altered in the renal cortex of *db/db* mice, we measured the mRNA expression levels of different ACSL isoforms. We found that *Acsl1* mRNA and ACSL1 protein expression were significantly increased in the proximal tubules of *db/db* mice as compared with *db/+* mice (Fig. 2, *A*–*C*). However, the mRNA expression of *Acsl6* was decreased in the *db/db* renal cortex (Fig. 2*C*). The *Acsl3*, *Acsl4*, and *Acsl5* mRNA expression levels were not significantly different in *db/db mice* as compared with *db/+* mice (Fig. 2*C*).

### PA induced *Acsl1*, *Acsl3,* and *Acsl5* mRNA expression in HK2 cells, while TNF-α induced only *Acsl1* expression

**Figure 3:**
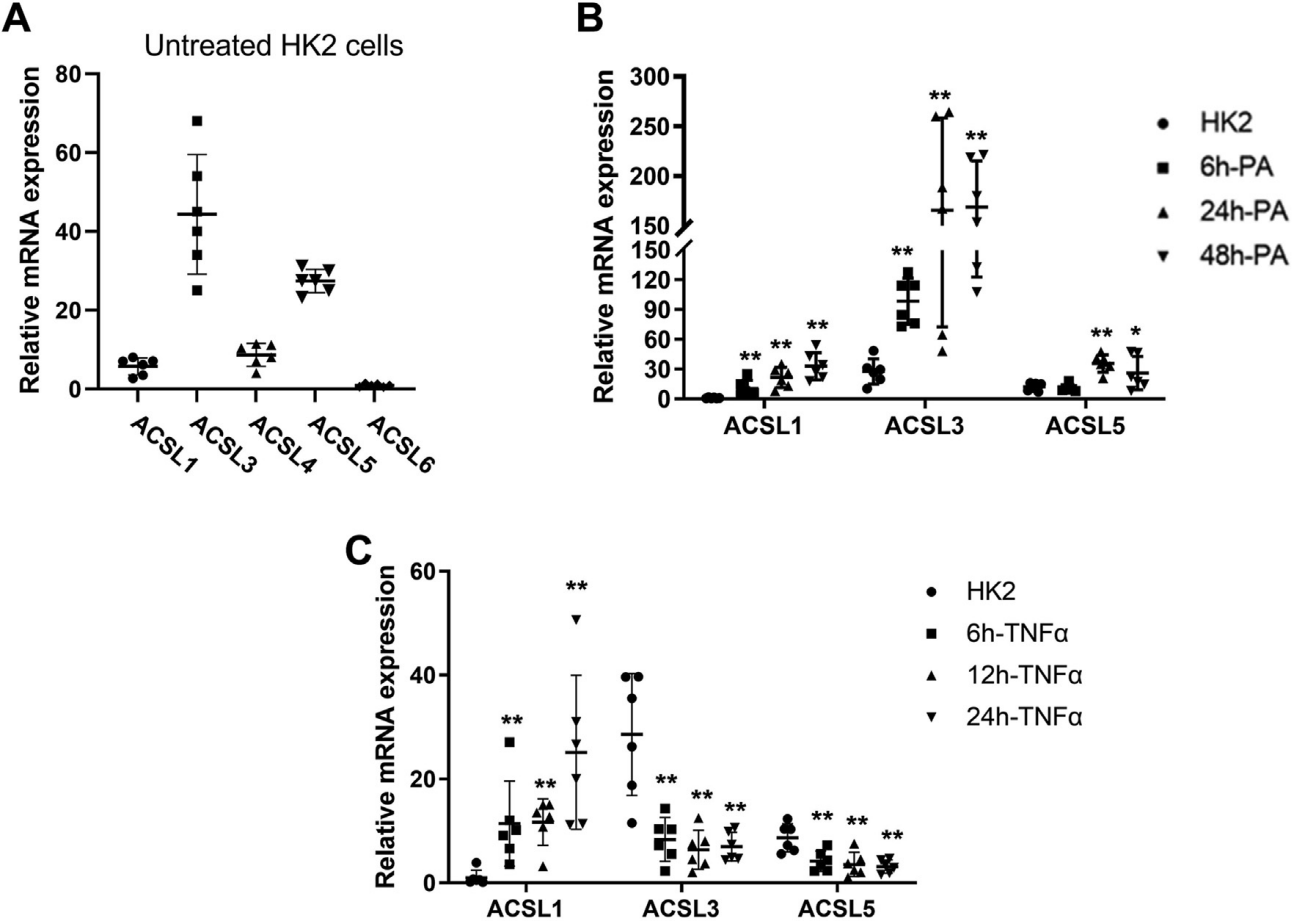
**Treatment with PA (saturated fatty acid) or TNF-α significantly induced *Acsl1* mRNA expression in HK2 cells, a human proximal tubule epithelial cell line.** To investigate the potential role for *Acsl* isoforms in renal lipid dysmetabolism and inflammation, characteristic features of DKD, we treated HK2 cells with either PA for 48 h or TNF-α for 24 h and measured the mRNA expression levels of *Acsl1* isoforms. *A,* mRNA expression of *Acsl1*, *Acsl3*, *Acsl4*, *Acsl5*, *Acsl6* in HK2 cells (n = 6). *B,* mRNA expression of ACSL isoforms, *Acsl1*, *Acsl3*, *Acsl5,* in HK2 cells treated with PA (200 mM; (16:0) bound to BSA) for 0, 6, 24, and 48 h (n = 6). *C,* mRNA expression of *Acsl1*, *Acsl3*, *Acsl5* in HK2 cells treated with TNF-α (10 ng/ml) for 0, 6, 12, and 24 h (n = 6). Statistical differences in mRNA expression levels were assessed using a two-tailed unpaired student’s *t* test. Data are shown as mean ± SD. ∗*p* < 0.05 and ∗∗*p* < 0.01. ACSL, acyl-CoA synthetase; BSA, bovine serum albumin; DKD, diabetic kidney disease; PA, palmitate; TNF, tumor necrosis factor.

Renal proximal tubule cells are energy-demanding cells and utilize fatty acid oxidation to generate the energy necessary to maintain renal functions, including sodium reabsorption and glucose metabolism (19, 20). We found that all *Acsl* isoforms were expressed in HK2 cells, with high gene expression of *Acsl3* and *Acsl5*, and very low gene expression of *Acsl6* (Fig. 3*A*).

To further investigate the potential roles for *Acsl* isoforms in renal lipid dysmetabolism and inflammation, characteristic features of DKD, we treated HK2 cells with either PA for 48 h or TNF-α for 24 h. PA-treated HK2 cells showed a significant time-dependent increase in *Acsl1*, *Acsl3*, and *Acsl5* mRNA expression with highest expression levels at 24- and 48-h posttreatment (Fig. 3*B*). There was a 20-fold increase in *Acsl1*, 5-fold increase in *Acsl3*, and 3-fold increase in *Acsl5* mRNA expression, following PA treatment (Fig. 3*B*). In contrast, TNF-α treatment increased only *Acsl1* mRNA expression in a time-dependent manner with highest expression at 24 h posttreatment and decreased *Acsl3* and *Acsl5* mRNA levels (Fig. 3*C*). These data suggest the important roles of *Acsl1*, *Acsl3,* and *Acsl5* in lipid metabolism, and a selective role of *Acsl1* in inflammation in the renal proximal tubular cells.

### PA- and TNF-α–induced responses in HK2 cells were abolished in cells with specific deletion of *Acsl1*

**Figure 4:**
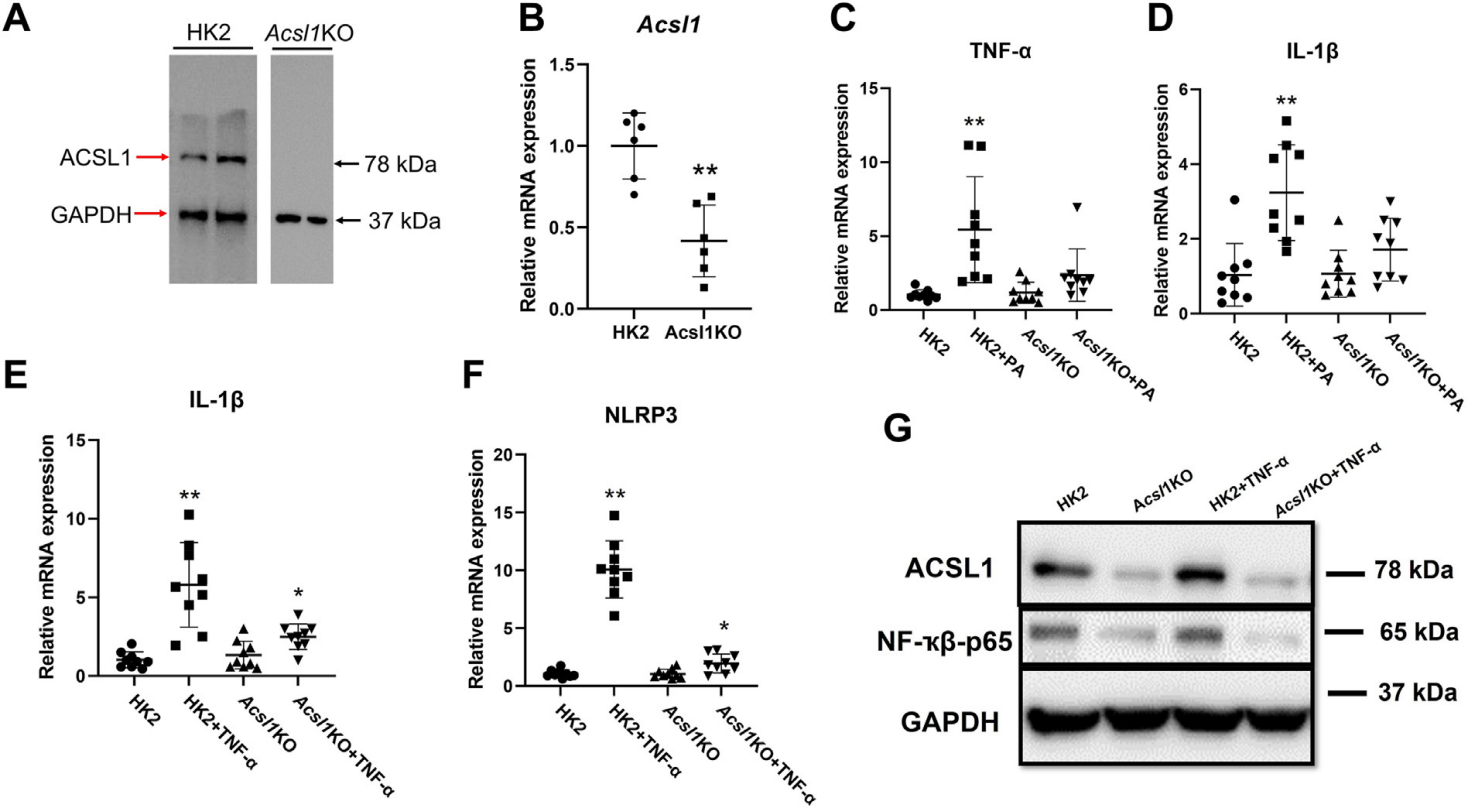
**ACSL1 plays a critical role in inflammation.** To examine the role of ACSL1 in lipid metabolism and inflammation, we generated HK2 cell lines with the specific deletion of *Acsl1* (*Acsl1*KO) using CRISPR/Cas9 genome editing. Control HK2 and *Acsl1*KO cells were treated with either PA (200 mM; (16:0) bound to BSA) or TNF-α (10 ng/ml) for 24 h. *A,* ACSL1 protein expression in HK2 and *Acsl1*KO cells (n = 6). *B, Acsl1* mRNA expression in HK2 and *Acsl1*KO cells (n = 9). *C* and *D,* IL-1β and TNF-α mRNA expression following PA treatment for 24 h (n = 9). *E* and *F,* IL-1β and NLRP3 mRNA expression following TNF-α treatment for 24 h (n = 9). *G,* NF-κβ protein levels in lysates assessed by Western blot (n = 3). Statistical differences in expression levels were assessed using a two-tailed unpaired student’s *t* test. Data are shown as mean ± SD. ∗*p* < 0.05 and ∗∗*p* < 0.01. ACSL, acyl-CoA synthetase; BSA, bovine serum albumin; NLRP, nucleotide-binding oligomerization domain, leucine-rich repeat-containing protein; PA, palmitate; TNF, tumor necrosis factor.

To further examine the role of ACSL1 in lipid metabolism and inflammation, we generated HK2 cell lines with the specific deletion of *Acsl1* (*Acsl1*KO) using CRISPR/Cas9 genome editing. After confirming a significant decrease in *Acsl1* mRNA and ACSL1 protein levels in the *Acsl1*KO cells (Fig. 4, *A* and *B*), *Acsl1*KO cells and control HK2 cells were treated with either PA or TNF-α for 24 h. PA treatment significantly increased IL-1β and TNF-α mRNA expression in HK2 cells, and these responses were abolished in *Acsl1*KO cells (Fig. 4, *C* and *D*). Similarly, TNF-α stimulation significantly induced IL-1β and nucleotide-binding oligomerization domain, leucine-rich repeat-containing protein 3 (NLRP3) mRNA expression in HK2 cells, while these responses were significantly reduced in *Acsl1*KO cells (Fig. 4, *E* and *F*). NF-kβ was also significantly increased in HK2 cells after TNF-α stimulation and its expression was decreased in *Acsl1*KO cells (Fig. 4*G*). These data further support the role of ACSL1 in the regulation of lipid metabolism and inflammation.

**Figure 5:**
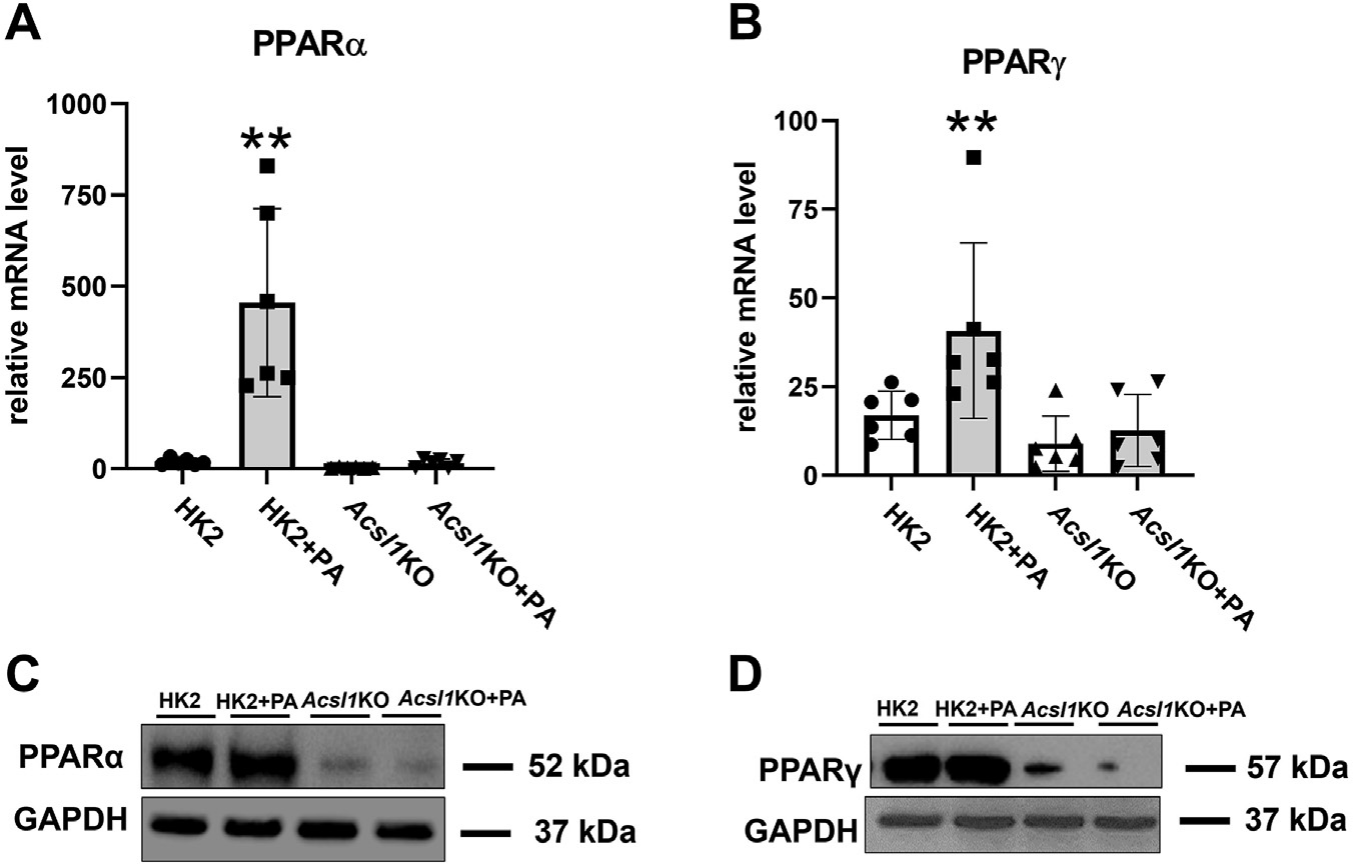
**ACSL1 regulates PPARα/γ-mediated control of lipid metabolism and inflammation in HK2 cells.** We examined the mRNA and protein expression levels of PPARα and PPARγ in HK2 and *Acsl1*KO cells. *A* and *B,* PPARα and PPARγ mRNA expression in HK2 and *Acsl1*KO cells treated with PA (200 mM; (16:0) bound to BSA) for 24 h. *C* and *D,* PPARα and PPARγ protein expression in lysate from HK2 and *Acsl1*KO cells treated with PA, assessed by Western blot. Statistical differences in expression levels were assessed using a two-tailed unpaired student’s *t* test. Data are shown as mean ± SD. ∗*p* < 0.05 and ∗∗*p* < 0.01. ACSL, acyl-CoA synthetase; BSA, bovine serum albumin; PA, palmitate; PPAR, peroxisome proliferator-activated receptor.

Because PPARα and PPARγ are involved in lipid metabolism and anti-inflammatory pathways and ACSL1 is a direct target gene of PPARα/γ in liver and adipose tissue (18, 19, 20, 21), we examined the expression levels of PPARα and PPARγ in HK2 and *Acsl1*KO cells. Twenty-four hours of PA treatment significantly increased PPARα and PPARγ mRNA and protein expression in HK2 cells, and this upregulation was abolished in *Acsl1*KO cells (Fig. 5). Taken together, these findings suggest a central role of ACSL1 in lipid metabolism and inflammation, possibly mediated by PPARα and PPARγ signaling pathways.

### PA- and TNF–α-induced alteration in acylcarnitines and acyl-CoAs in HK2 cells was abolished in *Acsl1*KO cells

**Figure 6:**
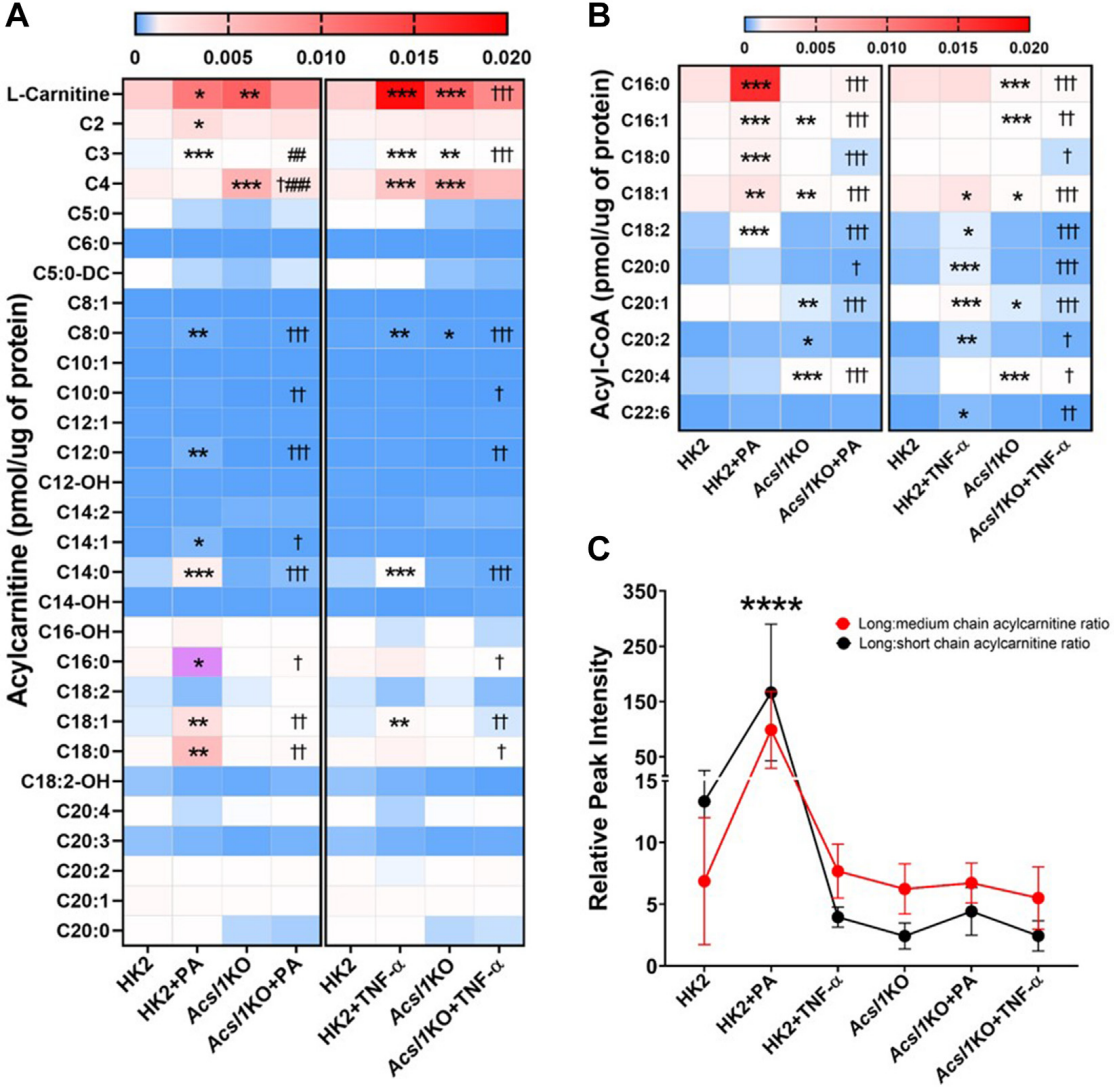
**ACSL1regulatesfatty acid β-oxidation through acyl-CoAs.** To investigate the role of ACSL1 in the regulation of fatty acid β-oxidation, we examined the levels of long-chain acylcarnitines and fatty acyl-CoAs in HK2 and *Acsl1*KO cells (n = 5/group), treated with either PA (200 mM; (16:0) bound to BSA), or TNF-α (10 ng/ml) for 24 h. Heatmap of concentrations of acylcarnitines (*A*) and acyl-CoAs (*B*) in HK2 and *Acsl1*KO cells treated with either PA or TNF-α. Statistical differences in acylcarnitines and acyl-CoAs concentration levels were assessed using a one-way ANOVA, followed by Tukey’s multiple comparisons test. ∗Significant difference from untreated HK2 cells, ∗*p* < 0.05, ∗∗*p* < 0.01, and ∗∗∗*p* < 0.001; ^†^Significant difference from PA- or TNF-α–treated HK2 cells, ^†^*p* < 0.05, ^††^*p* < 0.01, and ^†††^*p* < 0.001; ^#^Significant difference from *Acsl1*KO cells, ^##^*p* < 0.01, ^###^*p* < 0.001. *C,* comparison of the relative ratios of different length acylcarnitines in HK2 and *Acsl1*KO cells treated with either PA or TNF-α for 24 h. Statistical differences in relative peak intensity ratio of acylcarnitines between different groups were assessed using a two-way ANOVA, followed by Tukey’s multiple comparisons test. ^∗∗∗∗^HK2 cells treated with PA showed significantly higher ratio of long to medium and long to short chain acylcarnitines than other groups. Data are shown as mean ± SD. ACSL, acyl-CoA synthetase; PA, palmitate; TNF, tumor necrosis factor.

ACSL1 catalyzes the conversion of long-chain fatty acids to acyl-CoAs. The proinflammatory effects of saturated fatty acids are likely mediated by the acyl-CoA derivatives of the fatty acids (22). ACSL1 upregulates PPARα and PPARα further governs mitochondrial fatty acid β-oxidation mediated by long-chain acylcarnitines and acyl-CoAs by altering the expression of numerous target genes (22). To investigate the role of ACSL1 in the regulation of fatty acid β-oxidation, we examined the levels of long-chain acylcarnitines and fatty acyl-CoAs in HK2 and *Acsl1*KO cells treated with either PA or TNF-α for 24 h (Figs. 6, S1–S3, Tables S1, and S2). We found that levels of long-chain acylcarnitines and C16:0, C16:1, C18:0, C18:1, and C18:2 fatty acyl-CoAs were significantly increased in HK2 cells, following PA treatment. However, these responses were not observed in *Acsl1*KO cells (Fig. 6, *A* and *B*). In contrast, TNF-α treatment of HK2 cells significantly increased the levels of short-chain acylcarnitines and C18:1, C18:2, C20:0, C20:1, C20:2, and C22:6 fatty acyl-CoAs. These responses were again abolished in *Acsl1*KO cells (Fig. 6, *A* and *B*). Additionally, we found that the relative peak intensity ratios of acylcarnitines of different chain lengths (long-chain:short-chain and long-chain:medium-chain) were significantly increased in HK2 cells, but not in *Acsl1*KO cells, following PA treatment (Fig. 6*C*). In contrast, TNF-α treatment in HK2 cells significantly decreased the long-chain:short-chain ratio, with no effect on the long-chain:medium-chain ratio (Fig. 6*C*), suggesting impaired β-oxidation. These results demonstrate an important role of ACSL1 in modulating the fatty acid β-oxidation in response to PA- and TNF-α–induced inflammation in the renal proximal tubule epithelial cells.

## Discussion

Here, we report that ACSL1 modulates lipid metabolism and inflammation, major underlying causes of DKD development and progression, in the kidney proximal tubules. We observed this *in vivo* in the *db/db* mouse model as well as *in vitro* in a human proximal tubule epithelial cell line. Consistent with the previous studies, the *db/db* mice developed DKD by 24 weeks of age, accompanied by markedly increased renal fibrosis and inflammation (18, 23). We found significantly increased *Acsl1* mRNA and ACSL1 protein levels in the proximal tubules of *db/db* mice and in response to PA or TNF-α treatment of the HK2 cells. Further, increased expression of ACSL1 was accompanied by increased levels of the long-chain fatty acids required for subsequent mitochondrial β-oxidation. *In vitro* studies in *Acsl1*KO cells demonstrated that altered expression of inflammatory mediators and fibrosis-related genes and altered fatty acid acyl-CoA and acylcarnitine profiles in response to PA or TNF-α treatment were ACSL1-dependent. Previous studies have shown an important role for ACSL1 in lipid metabolism in liver and adipose tissue (19, 20, 24). Interestingly, our findings in this study indicate that ACSL1 plays an important role in lipid metabolism in the renal proximal tubules as well.

Treatment of HK2 cells with the fatty acid PA increased *Acsl1* mRNA expression 20-fold, more than the other ACSL isoforms, suggesting selective importance of ACSL1 in the regulation of lipid metabolism in renal tubule cells. Moreover, renal proximal tubule cell-specific *Acsl1* deletion significantly reduced the levels of long-chain fatty acyl CoAs and decreased the expression of proinflammatory cytokines and intracellular NF-κβ. This suggests that ACSL1 is required for long-chain fatty acids to stimulate the expression of proinflammatory cytokines in the renal proximal tubules. Long-chain fatty acids influence inflammation through a variety of mechanisms including translocation to the nucleus (25) and cell surface and intracellular receptors that control inflammatory cell signaling and gene expression (26). Further, saturated fatty acids stimulate inflammation mediated by toll-like receptor 4 (TLR4), a receptor for bacterial lipopolysaccharide (21). TLR4 plays a key role in innate immune responses through the NF-κβ/NLRP3 inflammasome signaling pathway (24). Importantly, our data suggest a tissue-specific response unique to renal tubule cells. Future studies will be needed to better define this pathway and to determine if there is an association between ACSL1 and TLR4 signaling.

Lipotoxicity has been implicated in the pathogenesis of DKD (27). Growing evidence suggests that lipotoxicity-induced renal damage depends not only on the quantity of lipids that accumulate in the kidney but also on the type of lipids (28). However, the specific contribution of dysregulated metabolism in the renal proximal tubules to the pathogenesis and progression of DKD has been largely unexplored. Our study addressed this question. We found that PA treatment significantly increased the levels of long-chain acylcarnitines and C16:0, C16:1, C18:0, C18:1, and C18:2 fatty acyl-CoAs in HK2 cells, and this regulation was ACSL1-dependent. In contrast, TNF-α treatment significantly increased the levels of L-carnitine, short-chain acylcarnitines, and C18:1, C18:2, C20:0, C20:1, C20:2, and C22:6 fatty acyl-CoAs in HK2 cells; these effects were also ACSL1-dependent.

Long-chain saturated fatty acids serve as energy sources, components of cell membranes, and precursors for signaling molecules, and several lines of evidence have implicated long-chain saturated fatty acids in mediating proinflammatory effects (29). Long-chain saturated fatty acids activate TLR4 signaling, which not only induces local inflammatory cytokine expression, but also induces stress responses in the endoplasmic reticulum (30). Moreover, arachidonic acid, a 20-carbon-chain fatty acid, activates TLR4, and loss of TLR4 function completely inhibits arachidonic acid induction of proinflammatory cytokines (31, 32). Thus, long-chain saturated fatty acids may act predominantly through TLR4 to induce the inflammatory responses that lead to the initiation and progression of DKD (33). Further studies are needed to validate and understand the role of long-chain saturated fatty acids in the pathogenesis of DKD.

ACSL1 may regulate lipid metabolism through PPARα/γ pathways. Several studies have shown that PPARα and PPARγ have antidiabetic effects and provide renal protection in DKD through energy metabolism, cell proliferation, and suppression of inflammation (34). We found that renal proximal-tubule-cell–specific *Acsl1* deletion significantly decreased PPARα and PPARγ mRNA and protein expression, suggesting an important role of ACSL1 in PPARα/γ-mediated protection against DKD.

Our study has some limitations. First, of the possible lipid mediators analyzed in this study, ACSL1 was identified as the most likely candidate involved in the inflammatory phenotype of DKD. However, we cannot rule out the possible contributions of other isoforms, such as ACSL3 and ACSL5. Second, renal proximal tubule metabolism is difficult to recapitulate in immortalized cell culture or isolated tubular culture systems. HK2 cells readily metabolize fatty acids, and therefore, findings in HK2 cells may not translate into *in vivo* models with modest fatty acid utilization. Mouse models of DKD with tubule-specific modulation of ACSL1 will be helpful to further elucidate the role of ACSL1 in renal proximal tubule physiology and in regulating lipid metabolism in DKD.

In conclusion, ACSL1 regulates inflammation and lipid metabolism in renal proximal tubule epithelial cells. Our data demonstrate that ACSL1 plays an important role in the pathogenesis of DKD by regulating long-chain saturated fatty acid–induced inflammation. Therefore, ACSL1 may be a potential therapeutic target for DKD treatment.

## Experimental procedures

### Animal care and housing

Male C57BLKS/J (BKS) *db/+* (control) and BKS *db/db* mice were purchased from Jackson Laboratories (BKS.Cg-Dock7m +/+ Lepr^db/J^, Stock No: 000642) at 4 weeks of age. The *db/db* mice provide a well-established model of type 2 diabetes. Animals were maintained in specific pathogen–free housing provided by the University of Michigan Unit for Laboratory Animal Medicine and were given access to water and standard chow *ad libitum*. All protocols were carried out in accordance with the guidelines outlined by the Diabetes Complications Consortium (http://www.diacomp.org) and the National Institutes of Health’s Guide for the Care and Use of Laboratory Animals (eighth edition). All protocols were approved by the University of Michigan Institutional Animal Care and Use Committee.

### Tissue and plasma collection

Twenty four–week-old *db/+* and *db/db* male mice were anesthetized using isoflurane anesthesia and perfused with sterile 0.9% saline through the abdominal aorta. The kidney cortex was dissected, placed in an embedding cassette, and immersed in paraformaldehyde solution overnight, followed by 70% ethyl alcohol (EtOH) in a beaker until tissue processing. Blood was collected from the inferior vena cava, and plasma was obtained using centrifugation at 4 °C for 20 min at 2000*g*.

### Masson’s trichrome staining

Sections (3-μm thick) from the renal cortex of *db/+* or *db/db* mice were processed and stained with Masson’s trichrome staining by The Tissue & Molecular Pathology Shared Resource at University of Michigan.

### ACSL1 immunofluorescence in proximal tubules in *db/db* mice

Renal cortex from *db/+* and *db/db* mice was dissected, embedded in paraffin and sectioned coronally at 3-μm thickness. Prior to staining, the slides were deparaffinized and rehydrated using the following protocol: xylene for 10 min, 100% EtOH for 5 min, 95% EtOH for 5 min, 70% EtOH for 5 min, and double-distilled water (ddH_2_O) for 5 min. The slides were then incubated in Retrieve-All-1 (100× stock, cat. # H-3300-250, Vector labs, diluted to 1× in ddH_2_O) at 95 °C for 2 h. After incubation, the slides were cooled for 10 min at room temperature, incubated in ddH_2_O water warmed to ∼37 °C for 10 min, and then rinsed in ddH_2_O (3 times, 5 min each). A border was drawn around each sample using a hydrophobic pen, and sections were processed for double-labeled immunofluorescence for ACSL1 and lotus tetragonolobus lectin (LTL, a proximal tubule marker). First, the sections were washed with 1× PBS; pH 7.4 (three times, 10 min each) and then treated with 2.5% normal donkey serum (cat. # D9663, Sigma-Aldrich) dissolved in 1× PBS containing 0.1% Triton X-100 (PBSDT) for 30 min to block nonspecific binding. Next, the sections were incubated overnight at room temperature with anti-ACSL1 primary antibody (dilution 1:50, cat. # 4047, Cell Signaling Technology) and fluorescein-labeled LTL (dilution 1:100; cat. # FL-1321-2, Vector Laboratories) in PBSDT. Thereafter, sections were washed 3 times in 1× PBS and incubated with Alexa Fluor 647–conjugated secondary antibody (dilution 1:200; donkey anti-rabbit, cat. # A-31573, Thermo Fisher Scientific) in PBSDT for 2 h at room temperature. Finally, sections were washed three times in 1× PBS for 10 min each and treated with a fluorescent stain, 4′,6-diamidino-2-phenylindole (cat. # R37606, Thermo Fisher Scientific), mounted using ProLong Gold Antifade mounting media (cat. # P10144, Thermo Fisher Scientific), cover slipped, and Alexa Fluor 647 (far red; ACSL1) and fluorescein (green; LTL) were visualized using a confocal microscope.

### Photography and image analysis

The images of renal cortex regions containing proximal tubules were captured using a Leica Stellaris 8 Falcon confocal microscope. The intensity of ACSL1-immunoreactivity was quantified in ten proximal tubules per mouse using Image J (https://imagej.nih.gov/ij/download.html, United States National Institutes of Health) and plotted as intensity/area.

### Cytokine quantitation

A multiplex assay for cytokines (cat. # MCYTOMAG-70K; Milliplex mouse cytokine panel, Millipore) was performed to measure IL-1β, IL-6, monocyte chemoattractant protein-1, and TNF-α in plasma samples (∼50 ul) by the Chemistry Core at University of Michigan. IL-18 ELISA was performed using a mouse IL-18 ELISA kit (cat. # 7625, MBL International Corporation) according to the manufacturer’s instructions. The kit had a high sensitivity of 25 pg/ml. All samples were run in duplicate.

### Cell culture

Human proximal tubule epithelial cells (HK2) and *Acsl1*KO cells were cultured at 37 °C in Dulbecco’s modified eagle medium/F12 medium containing 10% fetal bovine serum and 100 U/ml antibiotics cocktail (cat. # 15070063; Thermo Fisher Scientific) in a 5% CO_2_ incubator. Cells were split using trypsin after they reached 70 to 80% confluency and then cultured for another 24 h. At ∼70% confluency, cells were stimulated with either 200 mM PA (16:0) bound to bovine serum albumin (BSA) for 6, 24, and 48 h or 10 ng/ml TNF-α for 6, 12, and 24 h.

### CRISPR/Cas9 generation of human *Acsl1*KO cells

The GeneArt CRISPR Nuclease Vector with OFP Reporter Kit (cat. # A21174, Thermo Fisher Scientific) was used according to the manufacturer’s instructions to generate the HK2-*Acsl1*KO cells. A 20-bp target sequence of human ACSL1 (NM_001995; TACACCCTCTAATAAGAGTT) was used to design the *CRISPR* RNA–specific oligonucleotide primers. Equal molar amounts of each single-stranded oligonucleotide were annealed to generate a double-stranded oligonucleotide and ligated to the GeneArt CRISPR nuclease vector, then transformed into One Shot TOP10 chemically competent *Escherichia Coli*. The positively transformed clones were selected. DNA sequencing was used to confirm the GeneArt CRISPR Nuclease Vector construct. Empty vector transformants encoding GFP were used as negative controls.

Transfection was carried out when HK2 cells were 70 to 80% confluent in a 6-well plate. Lipofectamine 2000 transfection reagent (cat. # 11668027, Thermo Fisher Scientific) was used to transfect the CRISPR vector DNA (diluted 1:5 in dilution buffer) into the cells. After 48 h of culture, cells were harvested and washed so that they were in a single-cell suspension, and adjusted to a concentration of 1 × 10^6^ cells/ml in ice-cold fluorescence-activated cell sorting buffer (PBS, 0.5–1% BSA or 5–10% fetal bovine serum, 0.1% NaN3 sodium azide). Fluorescence-activated cell sorting was used to isolate the stably transfected *Acsl1*KO cells. Cells were cultured at 37 °C in a 5% CO_2_ incubator for further experiments.

### Acylcarnitine and acyl-CoA quantification

To quantify acylcarnitine and acyl-CoA levels, untreated and treated (PA or TNF-α) HK2 and *Acsl1*KO cells were plated at 10,000 cells/well in 6-well plates. Five wells for each group were used for acylcarnitine and acyl-CoA quantification, and one well was used for the total protein concentration estimation using a bicinchoninic acid protein assay. Cells in five wells were washed with 150 mM ammonium acetate, then 500 ul cold 8:1:1 methanol:chloroform:H_2_O was added to each well, and cells were harvested into Eppendorf tubes. The volume was divided into two equal parts; 250 ul was used for acylcarnitine quantification, and 250 ul was used for acyl-CoA quantification. For the acylcarnitine assays, the samples were spiked with a 1 ul of a cocktail of eight standards: L-carnitine, 152 pmoles; O-acetyl-L-carnitine, 38 pmoles; O-propionyl-L-carnitine, 7.6 pmoles; O-butyryl-L-carnitine, 7.6 pmoles; O-isovaleryl-L-carnitine, 7.6 pmoles; O-octanoyl-L-carnitine, 7.6 pmoles; O-myristoyl-L-carnitine, 7.6 pmoles; O-palmitoyl-L-carnitine, 15.2 pmoles (cat. # NSK-B-1, Cambridge Isotope Laboratories Inc.). For the acyl-CoA assays, each sample was spiked with 2 µl (20 pmoles) of an internal standard (C:17 CoA, heptadecanoyl CoA; Avanti Polar Lipids). The samples were mixed well, sonicated at pulse 80, power 3 for 2 min each, and mixed well again. The samples were kept on ice for 15 min, followed by centrifugation at 4 °C for 10 min at 14,000 rpm. The supernatant was collected and dried using either a vacuum concentrator at 45 °C (for acylcarnitine) or nitrogen gas (for acyl-CoA). Samples were then reconstituted in 40 ul of 95:5 H_2_O:acetonitrile in 15 mM ammonium hydroxide for acyl-CoA and 5 mM ammonium acetate in H_2_O for acylcarnitine. The samples were analyzed by LC-MS for the quantification of acylcarnitine and acyl-CoA. Values were normalized to the total protein of each sample and plotted as pmol/ug of protein for each sample.

### Western blotting

Protein lysates were prepared 48 h after transfection, and the protein concentration in the samples was determined using bicinchoninic acid protein assay kit. Total protein (30 ug) from cell lysates was denatured at 95 °C for 5 min. The proteins were resolved by SDS-PAGE and were transferred to a polyvinylidene fluoride membrane for 1 h at 200 mA voltage. The membrane was incubated in blocking buffer (5% skimmed milk powder in Tris-buffered saline with 0.1% Tween 20 (TBST) for 1 h at room temperature. Afterward, the membrane was incubated with primary antibodies for ASCL1 (cat. # 10585, Cell Signaling Technology), NF-κB p65 (cat. # 8242, Cell Signaling Technology), PPARα (cat. # ab233078, Abcam), or PPARγ (cat. # 2435, Cell Signaling Technology) overnight at 4 °C. All primary antibodies were diluted 1:1000 in 5% BSA. Washes were performed with 1× TBST. After washing, the membrane was incubated with the secondary antibody, horseradish peroxidase–linked antirabbit immunoglobulin G (cat. # 7074, Cell Signaling Technology), diluted 1:3000 in 1× TBST at room temperature for 1 h. The signal was visualized using a chemiluminescent imaging system with a 20 min exposure.

### Real-time polymerase chain reaction

Total RNA was extracted using the Purelink RNA Mini Kit (cat. # 12183020, Thermo Fisher Scientific). The quality of the RNA samples was tested using agarose gel electrophoresis. The RNA concentration and purity were tested using a NanoDrop 2000 spectrophotometer (Thermo Fisher Scientific). Complementary DNA was synthesized using SuperScript III Reverse Transcriptase kit (cat. # 18080044, Thermo Fisher Scientific). Quantitative RT-PCR was performed using SYBR Premix Ex Taq (cat. # A46012, Thermo Fisher Scientific). PCR was performed in triplicate to detect the transcription levels of *Acsl1, Acsl3, Acsl4, Acsl5, Acsl6, Il-1β*, *tnf-α*, *tgf-β*, connective tissue growth factor, *nlrp3*, *PPARA,* and *PPARG* (Table S3). β-actin was used as a reference gene for normalizing the mRNA levels. The PCR cycling conditions were 50 °C for 2 min, 95 °C for 10 min, 35 cycles of 95 °C for 10 s, 60 °C for 30 s, and 72 °C for 15 s. The threshold cycle value of each sample was the average SD of the triplicate samples, and the results were quantified using the 2-^ΔΔ^CT method.

### Statistical analysis

Statistical analyses were performed using GraphPad Prism 11 (https://www.graphpad.com, GraphPad Software). Two experimental groups were compared using two-tailed student’s unpaired *t* test. In experiments with multiple groups, one-way or two-way ANOVA, followed by Tukey’s post hoc correction was used to test the differences between groups. *p* < 0.05 was considered as statistically significant.

## Data availability

All primary data are available freely in the laboratory.

## Compliance with ethical standards

All applicable international, national, and/or institutional guidelines for the care and use of animals were followed.

## Supporting information

This article contains supporting information.

## Conflict of interest

The authors declare that they have no conflicts of interest with the contents of this article.
