# Supplementary material for: Fatty acids and inflammatory stimuli induce expression of long-chain acyl-CoA synthetase 1 to promote lipid remodeling in diabetic kidney disease
Source: J Biol Chem. 2023 Nov 26;300(1):105502. doi: 10.1016/j.jbc.2023.105502 (PMC10770716; doi:10.1016/j.jbc.2023.105502)
Supplement: Supplemental data [file mmc1.docx]

**Supplemental Data**

**Fatty acids and inflammatory stimuli induce expression of long-chain acyl-CoA synthetase 1 to promote lipid remodeling in diabetic kidney disease**

Chih-Hong Wang*^1,2,3^ Surbhi*^3^, Sayhaan Goraya^3^, Jaeman Byun^3^, Subramaniam Pennathur^3,4^

^1^Department of Physiology, Tulane University of School Medicine; ^2^Tulane Hypertension & Renal Center of Excellence, New Orleans, LA70112; ^3^Division of Nephrology, Department of Internal Medicine; ^4^Department of Molecular and Integrative Physiology, University of Michigan, Ann Arbor, Michigan 48105

1. **Supplemental** **Figure S1**
2. **Supplemental** **Figure S2**
3. **Supplemental** **Figure S3**
4. **Supplemental** **Table S1**
5. **Supplemental** **Table S2**
6. **Supplemental Table S3**

**Figure S1**


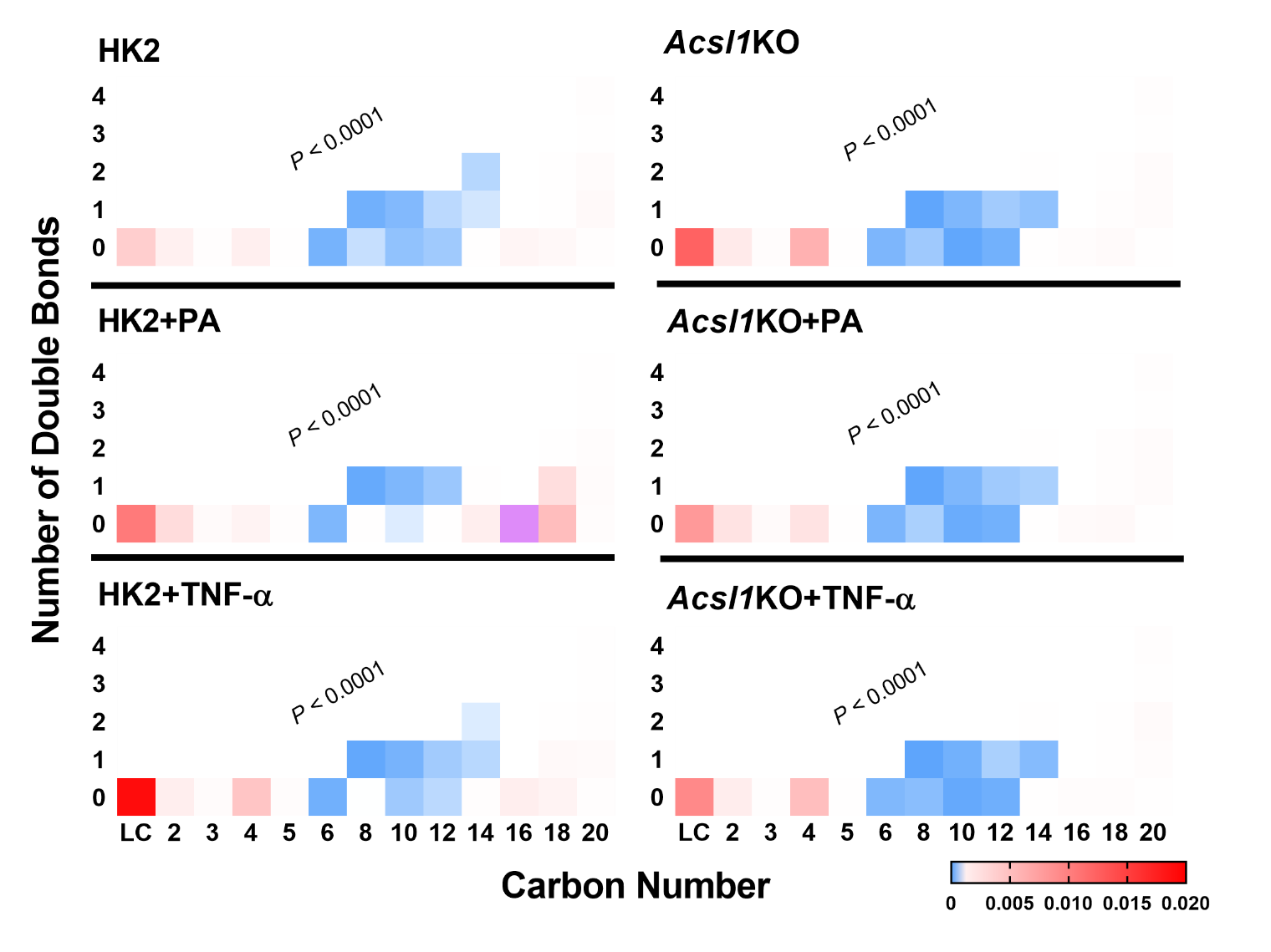


**Figure S1:** Comparison of standardized mean values of different acylcarnitines by carbon number and number of double bonds in HK2 and *Acsl1*KO cells (n=5/group), treated with (200 mM; (16:0) bound to BSA) or TNF-α (10 ng/mL). Statistical differences were assessed using one-way ANOVA followed by Tukey’s multiple comparisons test.

**Figure S2**

**
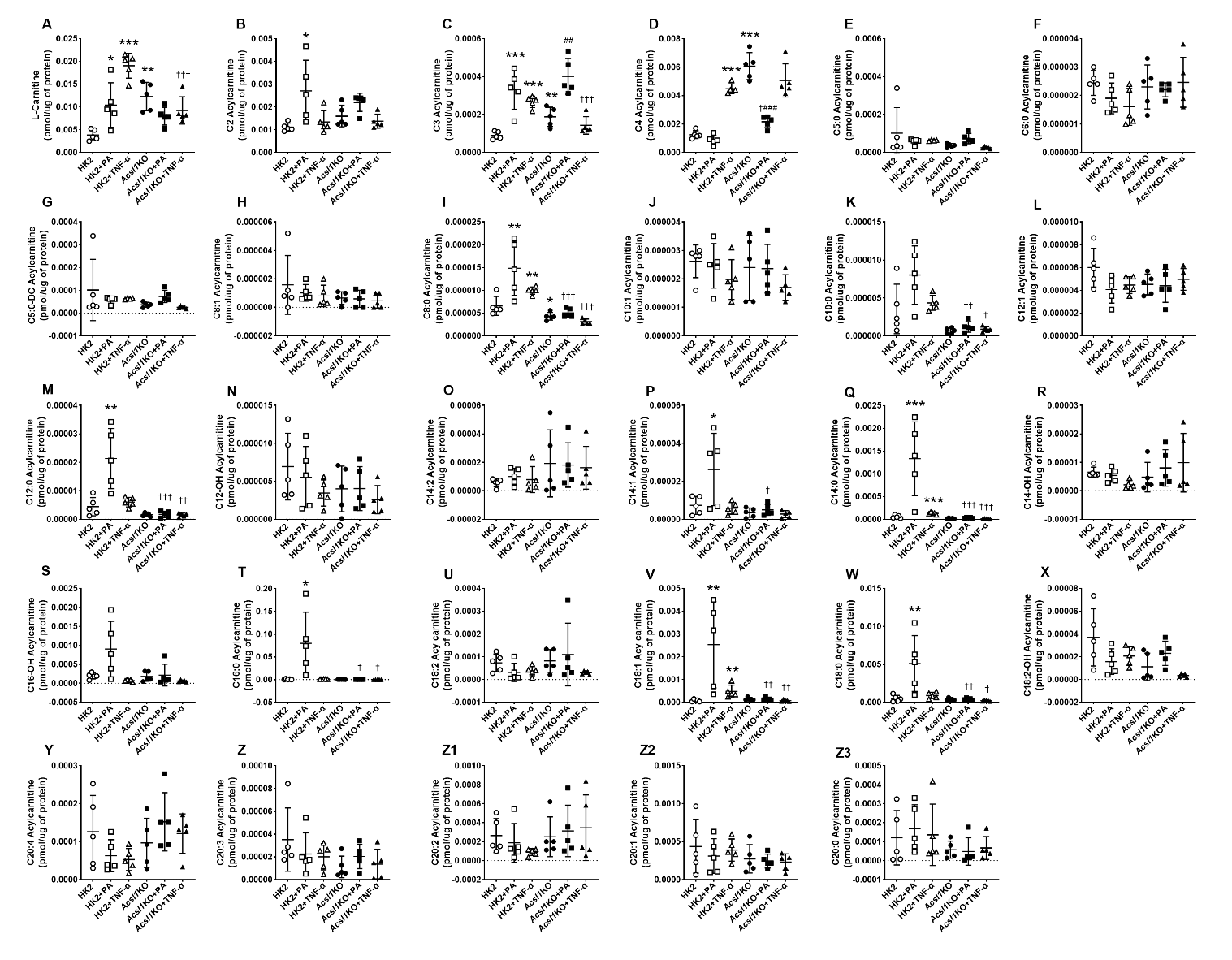
**

**Figure S2:** Concentrations of acylcarnitines with different chain lengths in HK2 and *Acsl1*KO cells (n=5/group), treated with either (200 mM; (16:0) bound to BSA) or TNF-α (10 ng/mL) for 24 hours. Statistical differences in acylcarnitines concentration levels were assessed using a one-way ANOVA followed by Tukey’s multiple comparisons test. ^*^Significant difference from untreated HK2 cells, ^*^p<0.05, ^**^p<0.01, ^***^p<0.001; ^†^Significant difference from PA or TNF-α treated HK2 cells, ^†^p<0.05, ^††^p<0.01, ^†††^p<0.001; ^#^Significant difference from *Acsl1*KO cells, ^##^p<0.01, ^###^p<0.001. Data are shown as mean ± SD.

**Figure S3**


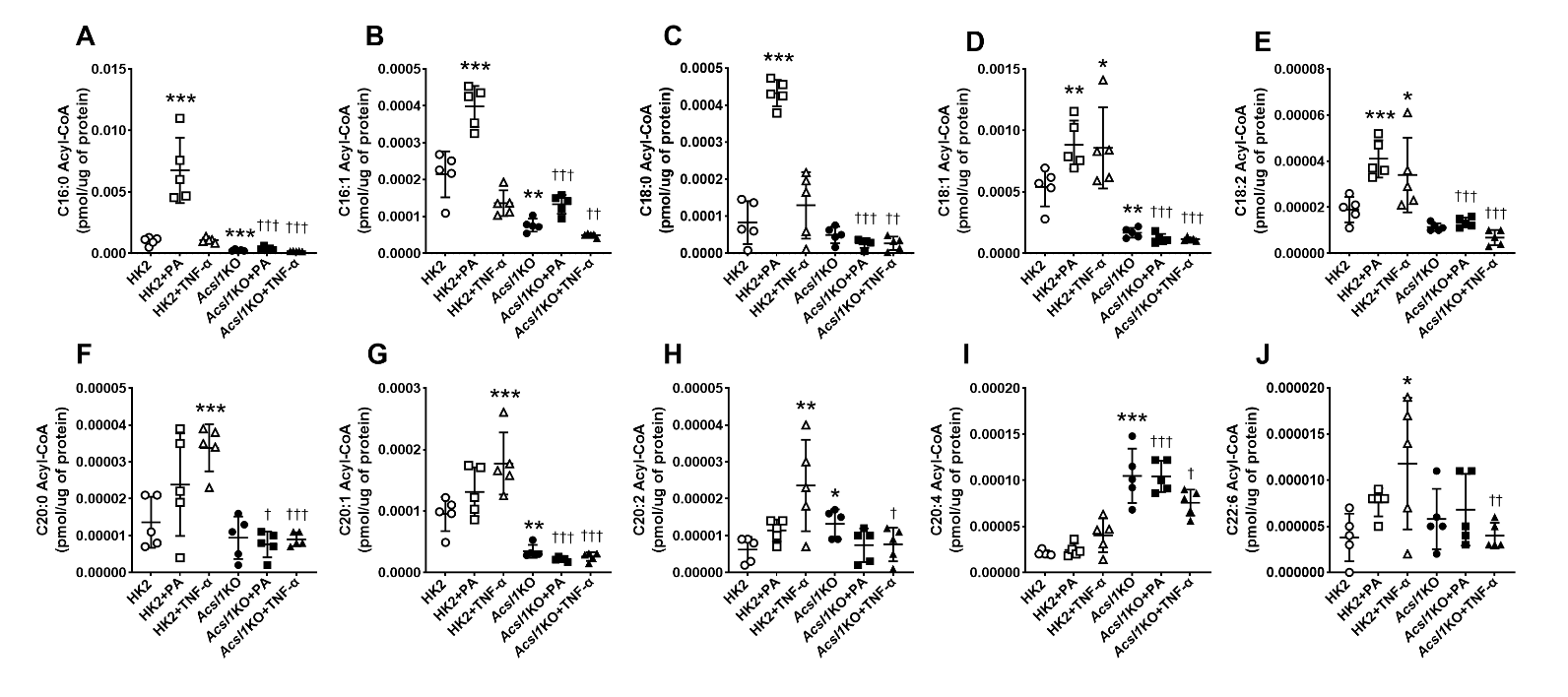


**Figure S3:** Concentrations of acyl-CoAs with different chain lengths in HK2 and *Acsl1*KO cells (n=5/group), treated with either (200 mM; (16:0) bound to BSA) or TNF-α (10 ng/mL) for 24 hours. Statistical differences in acyl-CoAs concentration levels were assessed using a one-way ANOVA followed by Tukey’s multiple comparisons test. ^*^Significant difference from untreated HK2 cells, ^*^p<0.05, ^**^p<0.01, ^***^p<0.001; ^†^Significant difference from PA or TNF-α treated HK2 cells, ^†^p<0.05, ^††^p<0.01, ^†††^p<0.001. Data are shown as mean ± SD.

**Table S1:** Concentrations of acylcarnitines (expressed as (fmol/ug of protein) in HK2 and *Acsl1*KO cells (n=5/group), treated with either (200 mM; (16:0) bound to BSA) or TNF-α (10 ng/mL) for 24 hours. Data are shown as mean ± SD.

| **Carnitine** | **HK2** | **HK2+PA** | **HK2+TNF-α** | ***Acsl1*KO** | ***Acsl1*KO+PA** | ***Acsl1*KO+TNF-α** |
| --- | --- | --- | --- | --- | --- | --- |
| **L-Carnitine** | 3.81±1.20 | 10.40±4.90 | 19.05±2.73 | 12.28±3.10 | 7.98±1.95 | 9.22±3.00 |
| **C2** | 1.11±0.18 | 2.70±1.35 | 1.34±0.50 | 1.59±0.47 | 2.20±0.40 | 1.37±0.31 |
| **C3** | 0.09±0.02 | 0.33±0.10 | 0.26±0.03 | 0.18±0.05 | 0.40±0.10 | 0.14±0.05 |
| **C4** | 1.25±0.28 | 0.87±0.34 | 4.48±0.35 | 6.07±0.95 | 2.15±0.41 | 5.06±1.17 |
| **C5:0** | 0.10±0.13 | 0.05±0.01 | 0.17±0.24 | 0.03±0.01 | 0.07±0.03 | 0.02±0.005 |
| **C6:0** | 0.002±0.0005 | 0.001±0.0005 | 0.00±0.0006 | 0.002±0.0008 | 0.002±0.0002 | 0.002±0.0009 |
| **C5:0-DC** | 0.10±0.134 | 0.05±0.014 | 0.17±0.24 | 0.03±0.011 | 0.07±0.03 | 0.02±0.005 |
| **C8:1** | 0.001±0.0020 | 0.001±0.0006 | 0.0008±0.0008 | 0.0006±0.0004 | 0.0006±0.0006 | 0.0005±0.0005 |
| **C8:0** | 0.006±0.0021 | 0.01±0.0059 | 0.009±0.0008 | 0.004±0.0008 | 0.005±0.0010 | 0.003±0.0005 |
| **C10:1** | 0.002±0.0006 | 0.002±0.0008 | 0.002±0.0007 | 0.002±0.0011 | 0.002±0.0008 | 0.001±0.0004 |
| **C10:0** | 0.003±0.0033 | 0.008±0.0038 | 0.004±0.0010 | 0.0007±0.0004 | 0.001±0.0007 | 0.0009±0.0004 |
| **C12:1** | 0.006±0.0017 | 0.004±0.0012 | 0.004±0.0007 | 0.004±0.0009 | 0.004±0.0014 | 0.005±0.0010 |
| **C12:0** | 0.004±0.0032 | 0.02±0.011 | 0.006±0.002 | 0.001±0.0006 | 0.001±0.001 | 0.001±0.0007 |
| **C12-OH** | 0.007±0.004 | 0.005±0.004 | 0.003±0.002 | 0.004±0.003 | 0.004±0.003 | 0.002±0.002 |
| **C14:2** | 0.005±0.003 | 0.01±0.006 | 0.007±0.009 | 0.01±0.02 | 0.01±0.016 | 0.01±0.015 |
| **C14:1** | 0.007±0.005 | 0.02±0.02 | 0.005±0.003 | 0.003±0.003 | 0.005±0.003 | 0.002±0.002 |
| **C14:0** | 0.05±0.034 | 1.33±0.81 | 0.12±0.02 | 0.01±0.01 | 0.03±0.01 | 0.01±0.005 |
| **C14-OH** | 0.006±0.002 | 0.005±0.002 | 0.002±0.002 | 0.004±0.005 | 0.008±0.006 | 0.0099±0.01 |
| **C16-OH** | 0.19±0.08 | 0.90±0.73 | 0.07±0.02 | 0.18±0.13 | 0.21±0.29 | 0.06±0.02 |
| **C16:0** | 0.77±0.90 | 79.64±68.52 | 1.26±0.33 | 0.24±0.06 | 0.38±0.13 | 0.18±0.11 |
| **C18:2** | 0.07±0.04 | 0.03±0.04 | 0.03±0.02 | 0.08±0.05 | 0.10±0.14 | 0.03±0.008 |
| **C18:1** | 0.08±0.05 | 2.53±1.90 | 0.47±0.28 | 0.14±0.06 | 0.14±0.07 | 0.07±0.04 |
| **C18:0** | 0.49±0.42 | 5.11±3.68 | 0.90±0.41 | 0.40±0.15 | 0.41±0.12 | 0.22±0.08 |
| **C18:2-OH** | 0.03±0.03 | 0.01±0.01 | 0.02±0.008 | 0.01±0.01 | 0.02±0.01 | 0.003±0.001 |
| **C20:4** | 0.12±0.10 | 0.06±0.04 | 0.05±0.03 | 0.09±0.06 | 0.15±0.08 | 0.12±0.05 |
| **C20:3** | 0.03±0.03 | 0.02±0.02 | 0.02±0.01 | 0.01±0.01 | 0.02±0.01 | 0.01±0.01 |
| **C20:2** | 0.26±0.18 | 0.18±0.20 | 0.09±0.04 | 0.25±0.21 | 0.31±0.27 | 0.34±0.35 |
| **C20:1** | 0.43±0.36 | 0.31±0.23 | 0.38±0.15 | 0.27±0.19 | 0.24±0.09 | 0.23±0.11 |
| **C20:0** | 0.12±0.14 | 0.16±0.13 | 0.13±0.16 | 0.05±0.05 | 0.04±0.07 | 0.06±0.06 |

**Table S2:** Concentrations of acyl-CoAs (expressed as fmol/ug of protein) in HK2 and *Acsl1*KO cells (n=5/group), treated with either (200 mM; (16:0) bound to BSA) or TNF-α (10 ng/mL) for 24 hours. Data are shown as mean ± SD.

| **Acyl-CoA** | **HK2** | **HK2+PA** | **HK2+TNF-α** | ***Acsl1*KO** | ***Acsl1*KO+PA** | ***Acsl1*KO+TNF-α** |
| --- | --- | --- | --- | --- | --- | --- |
| **C16:0** | 2.28±0.72 | 16.48±6.50 | 2.64±0.49 | 0.57±0.16 | 0.94±0.31 | 0.418±0.04 |
| **C16:1** | 0.48±0.14 | 0.97±0.14 | 0.33±0.09 | 0.17±0.04 | 0.31±0.06 | 0.11±0.012 |
| **C18:0** | 0.19±0.13 | 1.05±0.09 | 0.31±0.22 | 0.11±0.05 | 0.06±0.03 | 0.06±0.04 |
| **C18:1** | 1.23±0.36 | 2.15±0.48 | 2.10±0.81 | 0.38±0.09 | 0.28±0.09 | 0.27±0.04 |
| **C18:2** | 0.04±0.012 | 0.10±0.020 | 0.08±0.040 | 0.02±0.004 | 0.03±0.005 | 0.01±0.008 |
| **C20:0** | 0.03±0.015 | 0.05±0.035 | 0.08±0.015 | 0.02±0.013 | 0.01±0.009 | 0.02±0.004 |
| **C20:1** | 0.21±0.06 | 0.32±0.10 | 0.43±0.13 | 0.07±0.03 | 0.05±0.01 | 0.06±0.02 |
| **C20:2** | 0.01±0.008 | 0.02±0.007 | 0.05±0.03 | 0.03±0.009 | 0.01±0.011 | 0.01±0.011 |
| **C20:4** | 0.04±0.006 | 0.06±0.02 | 0.09±0.05 | 0.24±0.07 | 0.25±0.04 | 0.18±0.03 |
| **C22:6** | 0.009±0.006 | 0.01±0.004 | 0.02±0.002 | 0.01±0.008 | 0.01±0.009 | 0.005±0.001 |

**Table S3:** Real-time PCR primers sequences for genes of interest.

| **Target gene** | **Species** | **Accession no.** | **Primer Sequence (5'-3')** |
| --- | --- | --- | --- |
| *ACSL1* | Human | NM_001995 | Forward Primer - CCATGAGCTGTTCCGGTATTT  Reverse Primer - CCGAAGCCCATAAGCGTGTT |
| *ACSL3* | Human | NM_004457 | Forward Primer - GCCGAGTGGATGATAGCTGC  Reverse Primer - ATGGCTGGACCTCCTAGAGTG |
| *ACSL4* | Human | NM_004458 | Forward Primer - CATCCCTGGAGCAGATACTCT  Reverse Primer - TCACTTAGGATTTCCCTGGTCC |
| *ACSL5* | Human | NM_203379 | Forward Primer - CTCAACCCGTCTTACCTCTTCT  Reverse Primer - GCAGCAACTTGTTAGGTCATTG |
| *ACSL6* | Human | NM_001205248 | Forward Primer - GCACGGCGATCTGTGATTG  Reverse Primer - GGCGGAACACCTGGTACAT |
| *PPARα* | Human | NM_005036 | Forward Primer - ATGGTGGACACGGAAAGCC  Reverse Primer - CGATGGATTGCGAAATCTCTTGG |
| *PPARγ* | Human | NM_138711 | Forward Primer - ACCAAAGTGCAATCAAAGTGGA  Reverse Primer - ATGAGGGAGTTGGAAGGCTCT |
| *NLRP3* | Human | NM_001127462 | Forward Primer - GATCTTCGCTGCGATCAACAG  Reverse Primer - CGTGCATTATCTGAACCCCAC |
| *TNF-α* | Mouse | NM_013693.3 | Forward Primer - GGTGCCTATGTCTCAGCCTCTT  Reverse Primer - GCCATAGAACTGATGAGAGGGAG |
| *IL-1β* | Mouse | NM_008361 | Forward Primer - TGGACCTTCCAGGATGAGGACA  Reverse Primer - GTTCATCTCGGAGCCTGTAGTG |
| *TGF-β* | Mouse | NM_025729 | Forward Primer - ATGGCGCAAAACAGTCCACA  Reverse Primer - TGTAACATGCACTGGGATACCA |
| *CTGF* | Mouse | NM_010217 | Forward Primer - GGCCTCTTCTGCGATTTCG  Reverse Primer - GCAGCTTGACCCTTCTCGG |
